# Recurrent mutations promote widespread structural and functional divergence of MULE-derived genes in plants

**DOI:** 10.1093/nar/gkab932

**Published:** 2021-11-02

**Authors:** Geun Young Chae, Woo-Jong Hong, Min Jeong Jang, Ki-Hong Jung, Seungill Kim

**Affiliations:** Department of Environmental Horticulture, University of Seoul, Seoul 02504, Republic of Korea; Graduate School of Biotechnology and Crop Biotech Institute, Kyung Hee University, Yongin 17104, Republic of Korea; Department of Environmental Horticulture, University of Seoul, Seoul 02504, Republic of Korea; Graduate School of Biotechnology and Crop Biotech Institute, Kyung Hee University, Yongin 17104, Republic of Korea; Department of Environmental Horticulture, University of Seoul, Seoul 02504, Republic of Korea

## Abstract

Transposable element (TE)-derived genes are increasingly recognized as major sources conferring essential traits in agriculturally important crops but underlying evolutionary mechanisms remain obscure. We updated previous annotations and constructed 18,744 FAR-RED IMPAIRED RESPONSE1 (FAR1) genes, a transcription factor family derived from Mutator-like elements (MULEs), from 80 plant species, including 15,546 genes omitted in previous annotations. In-depth sequence comparison of the updated gene repertoire revealed that FAR1 genes underwent continuous structural divergence via frameshift and nonsense mutations that caused premature translation termination or specific domain truncations. CRISPR/Cas9-based genome editing and transcriptome analysis determined a novel gene involved in fertility-regulating transcription of rice pollen, denoting the functional capacity of our re-annotated gene models especially in monocots which had the highest copy numbers. Genomic evidence showed that the functional gene adapted by obtaining a shortened form through a frameshift mutation caused by a tandem duplication of a 79-bp sequence resulting in premature translation termination. Our findings provide improved resources for comprehensive studies of FAR1 genes with beneficial agricultural traits and unveil novel evolutionary mechanisms generating structural divergence and subsequent adaptation of TE-derived genes in plants.

## INTRODUCTION

Transposable elements (TEs) are a major driving force of genome evolution, affecting genome size and structure as well as gene regulation (1). TEs often generate novel genes that generally consist of protein-coding sequences flanked by repeat sequences (2,3), such as *DAYSLEEPER* [encoding an hAT transposase with terminal inverted repeats and important for angiosperm development (4)] and the *L* gene [with flanking long terminal repeats provides resistance in peppers against *Tobamovirus* species (5)]. Most TE-derived genes form multi-copy gene families by TE-mediated duplication and subsequently adapt through various evolutionary processes, followed by acquisition of diversified functions (5–8). These novel genes are increasingly recognized as important sources of beneficial agricultural traits and adaptive functions in plants in response to changing environments (8,9). However, detailed adaptation mechanisms of these gene families are yet to be understood.

The FAR-RED IMPAIRED RESPONSE1 (FAR1) gene family, including FAR-RED ELONGATED HYPOCOTYLS3 (FHY3), FAR1-related sequences (FRS) and FAR1-related sequence-related factors (FRF), is a group of major Mutator-like element (MULE)-derived genes in plants thought to have evolved to adapt to changing light conditions (8,10,11). FAR1 genes generally have an N-terminal FAR1 DNA-binding domain, central MULE transposase domain, and C-terminal SWIM zinc-binding domain (11). Most commonly studied in *Arabidopsis*, FAR1 is known to activate FAR-RED-ELONGATED HYPOCOTYL1 (FHY1) and FHY1-like (FHL), which subsequently induce light-controlled physiological processes of agricultural interest (8,12). In addition to *Arabidopsis*, other major agricultural crops, such as rice and wheat, contain multiple copies of FAR1 genes; however, very little information regarding the functions of FAR1 genes in these species has been reported (13). Moreover, publicly available annotations with gene omissions limit a comprehensive understanding of the FAR1 gene family in plants, including important agricultural crops.

In this study, we performed annotation updates and consolidated 18,744 re-annotated FAR1 genes from 80 plant genomes. This updated gene repertoire comprised 15,546 genes previously omitted from annotations, especially from the *Poaceae* family of monocots, which includes many important crops such as wheat and rice. Comparative analyses of the top three gene structures of identified FAR1 genes revealed that premature translation termination caused by recurrent frameshift and nonsense mutations primarily led to the emergence of diversified gene structures. We identified a novel functional gene, encoding a fertility-related transcriptional regulator of pollen in *Oryza sativa* ssp. *japonica*, through knockout experiments using CRISPR/Cas9 and transcriptomic approaches. Further genomic investigation revealed that the functional gene emerged and adapted through a frameshift mutation generated by a tandem duplication of a 79-bp sequence specific to *O. sativa* ssp. *japonica* that led to premature translation termination. The updated FAR1 repertoire represents an important genetic source of acquired traits in plants and illustrates a dynamic adaptation mechanism for widespread structural and functional divergence that is mediated by a prominent TE-derived gene family. Moreover, our annotations provide a valuable genomic resource for future studies of beneficial agriculture traits, particularly regarding the genetic events that led to their emergence and phylogenetic distribution.

## MATERIALS AND METHODS

### Genomic resources and FAR1 gene family annotation

To annotate FAR1 family genes within the plant kingdom, 80 plant species, including 5 lower plants, 3 gymnosperms, 1 basal angiosperm, 35 monocots and 36 eudicots, were chosen for analyses (Supplementary Table S1). Of the total genes, those from 49 species were integrated from a previous study (14). Re-annotation of FAR1 genes in the remaining 31 species was performed in the same manner using TGFam-Finder v1.01 (14), publicly available genomic resources, and RNA-seq data described in Supplementary Table S1. TSV files obtained by performing InterproScan v5.22–61.0 (15) (-f tsv -appl Pfam) were used for ‘TSV_FOR_DOMAIN_IDENTIFICATION’, and the target domain ID was set as PF03101 (FAR1) from the PFAM database (16).

### Domain structures of FAR1 genes

Functional annotation of updated FAR1 gene models was completed with InterproScan v5.22–61.0 (15) (-f tsv -appl Pfam). SWIM zinc-finger domains in 18,744 protein sequences were identified by HMMER 3.1b2 (17) using default parameters and a Hidden Markov Model (HMM) database of SWIM (PF04434). Domain structures of each FAR1 gene were constructed from the modified InterproScan results, ignoring any domains with e-values higher than 1E-4. Only genes with at least one FAR1 (PF03101) domain were considered for downstream analyses. Domains excluding FAR1 (PF03101), MULE (PF10551) or SWIM (PF04434) were considered ‘integrated domains’ (IDs).

### Motif analysis of FAR1 genes

All protein sequences from FAR1 gene models were used as input for *de novo* motif discovery by MEME v5.1.1 (18) (-protein -mod zoops -nmotifs 100 -minw 10 -maxw 50 -objfun se -markov_order 0). A total of 79 motifs identified by MEME were then matched to 18,744 protein sequences using MAST v5.1.1 (19). Motifs appearing repetitively in various locations were excluded from defining motif position in the top three gene structures.

### Chi-square enrichment test

An in-house Perl script including fisher.test and chisq.test functions from the *Statistics::R* module in R were used to determine whether gene structures, ID, and motifs were enriched in monocots or eudicots. *P*-values were approximated by Monte Carlo simulations using 10,000 replicates for Fisher’s and chi-square tests to control familywise error rate and false discovery rate. P-values <0.0001 were considered highly significant for the confident enrichment test.

### Phylogenetic analyses of FAR1 genes

FAR1 genes with intact FAR1 domains containing the first and last motifs of FAR1 domains were used for phylogenetic tree construction. The 12,590 protein sequences were aligned by fftns from MAFFT v7.470 (20). Poorly aligned regions were calculated and removed by trimAl v1.4.rev22 (21) (-gappyout). RAxML v8.2.12 (22) predicted the PROTGAMMAJTTF model to be most suitable for this dataset (-m PROTGAMMAAUTO -p 12345), and 500 rapid bootstraps were run with random parsimony seeds to support the best maximum likelihood tree (-m PROTGAMMAJTTF -p 12345 -x 12345 -# 500). The tree was mid-point rooted and visualized using Interactive Tree of Life v5.7 (23), and the finalized tree was arranged into 28 subgroups based on FAR1 motif architectures and taxonomy. Intact FAR1 genes with similar domain and motif structures were grouped together, whereas those with distinct gene structures were further subdivided into separate groups. The remaining genes with partial FAR1 domains not included in the tree were assigned to specific subgroups by a BLASTP (-outfmt 7 -evalue 1E-10 -max_target_seqs 50) similarity search against all available intact FAR1 protein sequences. IQ-TREE v2.0.6 (24) (-mset JTT -mfreq F -alrt 1000 -B 1000 -safe) was used to infer phylogenetic relationships with the same dataset to validate the subgroup division of the tree from RAxML. Genes in each subgroup were examined to determine if they branched together in both trees.

### Identification of point mutation sites in FAR1 genes

DNA sequences of genes containing a FAR1 domain only (F) or FAR1 and MULE domains only (FM) were extended downstream by 40 and 20 kb, respectively. Then, they were searched with HMMER 3.1b2 (17) using default parameters and HMM databases of FAR1 (PF03101), MULE (PF10551) and SWIM (PF04434) to find evidence of MULE and/or SWIM domains in the downstream regions of F and FM genes. DupGen_finder (25) was used to define duplication pairs, which were grouped into two categories, depending on whether each pair had the same or different gene structures; the distinct domain architectures of the latter group were inspected. Between the two genes in a pair, the one with a shorter gene structure was designated as Pair 1, while the longer one was labeled Pair 2. The pairs were designated ‘F-type’ or ‘FM-type’ based on whether Pair 1 was an F or FM gene, respectively. Peptide sequences of Pair 2 were aligned to extended DNA sequences of Pair 1 using Exonerate v2.2.0 (26) (–model protein2genome –minintron [minimum intron length of Pair 2 * 0.9] –showtargetgff yes –showquerygff yes –codongapopen -1 –forcegtag yes). MAST v5.1.1 (19) was performed on the alignment using 79 previously identified motifs. All motifs in the alignment were grouped into four categories: front, pre-end, end and back (Figure 3A). The end motif containing a stop codon at the C-terminal of Pair 1 was labeled ‘end’ motif site and, the motif positioned in front of the ‘end’ motif site in the alignment was considered ‘pre-end’ motif site. Any motifs located upstream of the ‘pre-end’ motif site were classified as ‘front’ motifs, while motifs aligned downstream of the ‘end’ motif site were designated ‘back’ motifs.

Shortening of gene structures was expected to have occurred through one of two mechanisms: premature translation termination or MULE/SWIM domain truncation. If the ‘end’ motif site of Pair 1 was located immediately after the last domain of the protein sequence, the pair was predicted to experience premature translation termination. In contrast, if the ‘end’ motif site was positioned after a domain missing from the annotation, the sequence was thought to have undergone a partial truncation of its MULE/SWIM domains. Thus, the sequence variation of these two types was viewed separately. For noise filtration, only alignments covering >70% of Pair 2 were considered (Supplementary Figure S7). Additionally, for examining variation in sequences with premature translation termination, alignments containing all four categories of motifs (front, pre-end, end, back) were used for downstream analyses. For viewing sequence variation causing MULE/SWIM domain truncation, alignments containing remnants of MULE/SWIM domains were used (Supplementary Figure S7). Numbers of frameshift or nonsense mutations were counted for each motif position, with multiple mutations counted as one per motif position.

### Phylogenetic heatmap analysis

To choose a candidate for functional investigation in rice, we constructed a phylogenetic heatmap like that of the CAFRI-Rice website (http://cafri-rice.khu.ac.kr/) (27). Briefly, we measured the expression of FAR1 genes in the G2 subgroup using a modified gene annotation file (Dataset S1) and merged it with the phylogenetic tree of G2 subgroup genes through the ETE3 toolkit (28).

### RNA extraction and qRT-PCR analysis

Samples of *Oryza sativa* ssp. *japonica* were immediately frozen with liquid nitrogen at various developmental stages as described by Moon *et al.* (29). For pollen samples, we used RNAlater Stabilization Solution (Invitrogen) to collect and preserve pollen from dehiscent anthers at the paddy field. Total RNA was extracted using a RNeasy Plant Mini Kit (Qiagen) following the manufacturer’s instructions. cDNAs were synthesized using the SuPrimeScript RT Premix Kit (GeNet Bio) with a 50°C incubation for 60 min. For qRT-PCR analysis, we used 2X Prime Q-Mastermix which contains SYBR Green1, with PCR cycling conditions of 95°C for 10 s, 60°C for 15 s and 72°C for 20 s using the Rotor-Gene Q system (Qiagen). *Rice ubiquitin 5* (*OsUbi5*, *LOC_Os01g22490*) was used as a reference gene, and relative expression was calculated by the 2^−ΔΔCt^ method (30). The primers used for qRT-PCR are listed in Supplementary Table S9.

### Vector construction and rice transformation

To investigate subcellular localization, the coding sequences of *OSAT.v7_FAR1.Chr10.2* (hereafter called *OFF*) and *OsRH36* were amplified and fused with GFP and mCherry, respectively, in the HindIII-digested pGreen vector using the In-Fusion HD PCR Cloning Kit (Takara). The plasmids were transformed into *Agrobacterium tumefaciens* GV3101 individually and used for tobacco infiltration assays as described below. To generate a knockout mutant of *OFF* using CRISPR/Cas9 genome editing, we designed a single-guide RNA through CRISPRdirect (http://crispr.dbcls.jp/) (31). Designed oligomers were synthesized, and annealed oligomer was ligated into the BsaI-digested pRGEB32 binary vector (Addgene plasmid ID: 63142). After the plasmid was transformed into *A*. *tumefaciens* LBA4404, stable transformation of rice was performed using cv. Dongjin through the *Agrobacterium*-mediated co-cultivation method as described in Lee *et al.* (32). The primers for vector construction are listed in Supplementary Table S9.

### Subcellular localization assay

*Agrobacterium tumefaciens* GV3101 cells carrying the constructed p35S:OFF-GFP and p35S:OsRH36-mCherry vectors were co-infiltrated into *Nicotiana benthamiana* (tobacco) leaves following the protocol by Sparkes *et al.* (33). The infiltrated leaves were observed after 48–72 h with a confocal scanning laser microscope (LSM 510 META; Carl Zeiss). Fluorescence of GFP and mCherry was detected using 488/505–530- and 543/560–615-nm filter sets (excitation/emission), respectively. The acquired images were analyzed with Zeiss ZEN lite v2.6.

### Plant materials and growth conditions

Wild-type rice (*O. sativa* ssp. *japonica* cv. Dongjin) and the knockout mutant (*off-1*) were cultivated in a growth chamber under 28°C/25°C (day/night), 16 h/8 h light/dark cycle and 80% humidity conditions for 2 weeks and transferred to the paddy field located at Kyung Hee University in Yongin, South Korea, from May 2019 to October 2020. Seeds from these plants were grown on Murashige and Skoog media for 2 weeks and transferred to soil in the growth chamber. To obtain homozygous knockout mutants, genomic DNA was extracted using the CTAB method, and the CRISPR/Cas9 target region was amplified for Sanger sequencing analysis. Tobacco plants for the infiltration assay were cultivated in a growth chamber at 25°C with a 12 h/12 h light/dark cycle and 60% of humidity for 3 weeks.

### Transcriptome analysis

Anthers containing mature pollen grains were collected from the paddy field and immediately frozen with liquid nitrogen for RNA sequencing analysis. After passing quality control of the total extracted RNA, sequencing libraries were constructed using the TruSeq Stranded mRNA LT Sample Prep Kit following the manufacturer’s instructions (Part #15031047 Rev. E). RNA sequencing was performed by Macrogen, Inc., using the Illumina NovaSeq 6000 platform (Illumina). Raw FASTQ files were trimmed using Cutadapt v2.3 (34) (-a AGATCGGAAGAGC -A AGATCGGAAGAGC -q 30 -m 20) and mapped to the MSU7 reference rice genome (http://rice.plantbiology.msu.edu/) (35) using the HISAT2 aligner v2.1.0 with default parameters (36). The mapped reads were calculated using FeatureCount v1.6.3 (-t exon -g gene_id -p) (37) and statistically tested using DESeq2 v1.30.0 (38) in R. Differentially expressed genes (DEGs) were selected with the following criteria: basemean > 10, |log2(fold change)| > 1, and p- and adj.*P*-value < 0.05. To investigate FAR1 binding sites (FBS; CACGCGC) in 2-kb sequences located upstream of the DEGs, we parsed the upstream sequences using Bedtools v2.26.0 (39) and performed enrichment analysis of FBS through the AME function on the MEME Suite website (https://meme-suite.org/meme/tools/ame) (40). MapMan software was used for functional analysis of the DEGs between wild-type versus *off-1* mutant plants (41). The raw data files were deposited at ArrayExpress (https://www.ebi.ac.uk/arrayexpress; E-MTAB-10106).

### Phenotype analysis and fertility measurement

Flowers and anthers before anthesis were photographed with an SZX61 microscope (Olympus). To determine pollen grain morphology, each anther was squeezed with forceps, and the released pollen grains were stained as described in Kim *et al.* (42). Briefly, 1% I_2_-KI solution was used to stain the starch inside the pollen grain, and 0.1% calcofluor white and 0.001% auramine O solution were used to stain the intine and exine, respectively. The tri-stained pollen grains were observed using a BX61 microscope (Olympus) under brightfield, ultraviolet and fluorescein isothiocyanate channels, respectively. To observe plant phenotypes, homozygous plants and wild-type controls were grown in the paddy field and photographed using COOLPIX P900s (Nikon). Fertility was measured by calculating the seed setting ratios of five panicles from three wild-type and *off-1* knockout plants (six plants total) (Supplementary Table S10).

### Investigation of the emergence of functional gene

To elucidate the emergence of *OFF* in *O. sativa* ssp. *japonica*, recent ancestral gene structures of the functional gene were first identified by protein mapping of FMS-type genes in the same subgroup (G2) against chromosome 10 of other *Oryza* spp. using Exonerate v2.2.0 (26) (–model protein2genome –showtargetgff yes –showquerygff yes –forcegtag yes). Alignment results mapping >90% of *OSAT.v7_FAR1.Chr12.1* were considered to have a copy of the ancestral gene sequence with the FAR1-MULE-SWIM (FMS) structure. Mutation sites were detected in the alignment results. A multiple sequence alignment was performed using CLC Sequence Viewer 8 (CLC Bio), which identified an insertion of a 79-bp sequence in *OFF*.

### Yeast-two-hybrid screening assay

FAR1 domain coding sequence of *OFF* (2nd amino acid to 190th) was cloned into the EcoRI/BamHI-digested pGBKT7 vector. The self-transcriptional activation of the bait plasmid was examined by transforming into the AH109 yeast strain together with an empty prey vector. After confirming that there is no self-activation of baits, the yeast-two-hybrid screening assay was conducted by Panbionet Corp (Pohang, South Korea) using rice anther yeast library. Of the 8.88 × 10^6^ screened colonies, 60 colonies were grown on SD medium lacking leucine, tryptophan, histidine and adenosine (SD-LWHA) plates to find real positive interactions. To confirm the interaction, the prey part of DNA from 60 positive candidates were amplified by PCR or by *Escherichia coli* transformation, and then the amplified candidate prey was reintroduced into yeast with the FAR1 bait plasmid or with a negative control plasmid. The primers used for bait construction are listed in Supplementary Table S9.

## RESULTS AND DISCUSSION

### Structural characteristics of updated FAR1 genes among 80 plant species

**Figure 1.**
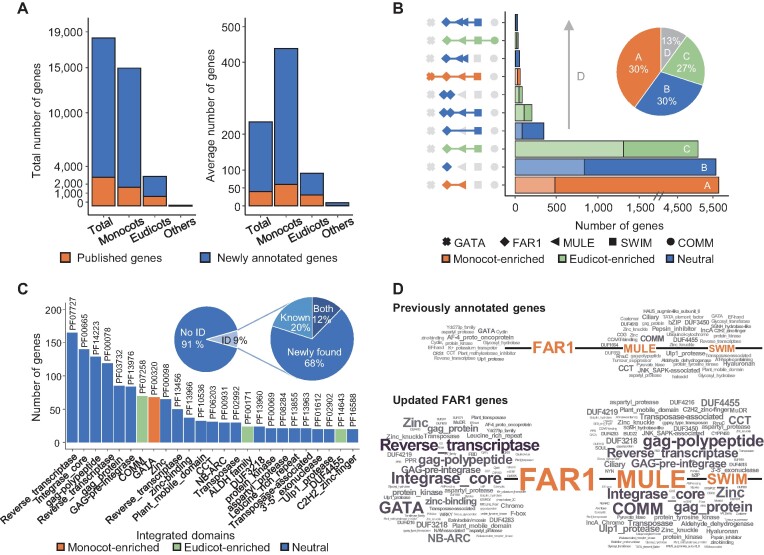
Characteristics of the re-annotated FAR1 gene family in 80 plant species. (**A**) Total and average numbers of FAR1 genes identified from 80 plant species, including 35 monocots and 36 eudicots, are illustrated as bar plots. Orange and blue bars indicate previously published genes and newly annotated genes, respectively. (**B**) The numbers of top 10 gene architectures found in the FAR1 genes are shown in bar plots, while the overall ratio of the top three structures and the others are presented in the pie chart. Different shapes on the left represent individual domains, and bar colors indicate taxonomic enrichment test results for each gene structure (*P* < 0.0001). Bars with lighter colors show numbers of published genes, whereas darker colors show newly annotated genes. (**C**) The numbers of genes with different integrated domains (IDs) are depicted as bar plots (top 25), and the overall proportion of genes containing IDs is displayed as pie charts. The pie chart on the right shows the proportion of IDs found in previously or newly annotated genes or both. Bars were colored according to taxonomic enrichment test results (*P* < 0.0001). (**D**) ID repertoires are depicted for published genes only and all genes, including those newly annotated in the current study. The approximate number of each ID is proportional to its size, and the major domains are highlighted in orange.

We performed re-annotation of FAR1 gene family (hereafter called FAR1 genes) in 31 plant species and consolidated a total of 18,744 updated genes from these and an additional 49 plant genomes by integrating previously improved FAR1 gene models (14) (Supplementary Table S1). Of the updated genes from 80 total species, 15,546 (83%) were newly annotated, which was a 5-fold increase from 3,198 (17%) previously annotated genes and were unevenly distributed across taxonomic classes: 82.1% (monocots), 17.5% (eudicots) and 0.4% (others) (Figure 1A and Supplementary Table S1). We observed that 10,693 (69%) of the newly annotated genes were generated based on RNA-Seq or protein data, showing high-confidence evidence (Supplementary Table S2). Most monocot FAR1 genes were predominantly observed in the *Poaceae* family (92%), suggesting a lineage-specific expansion of FAR1 genes that corroborates a report that *Mutator* sequences are ubiquitous in the grasses (43).

Structural analysis of FAR1 genes revealed that the order of abundance of three major gene structures (87%) was reversed after our annotation update; in contrast to 1,306 (40.8%), 833 (26.0%) and 478 (14.9%) previously annotated genes containing FAR1-MULE-SWIM (FMS), FAR1 only (F) and FAR1-MULE (FM), respectively, our updated gene models included 5,067 (27.0%), 5,559 (29.7%) and 5,634 (30.1%) genes with these respective elements (Figure 1B and Supplementary Table S3). Specifically, a chi-square test showed that FM genes were significantly abundant in newly annotated genes, especially among monocots, compared with prior analyses (Figure 1B and Supplementary Table S3). Considering the absence of known functional FM genes in model plants like *Arabidopsis*, our findings suggest that the updated annotation can serve as a novel resource for functional investigations of FAR1 genes (Supplementary Tables S3 and S4). Of the 2,484 genes (13%) excluding the three major gene architectures, 1,603 contained 570 distinct integrated domains (IDs) comprising high proportion of transposable element (TE)-related content, such as reverse transcriptase, integrase and gag polypeptide, closely associated with gene regions and function (44,45), as well as several lineage-specifically enriched domains, such as GATA and COMM (Figure 1C and D). Specifically, 68% of distinct IDs were identified in newly annotated genes and contributed to the annotation of new FAR1 gene structures (Figure 1C and D). These observations demonstrate that this updated annotation could provide improved FAR1 gene resources that contain a large number of previously non-annotated genes for further comprehensive analyses.

### Motif architectures of the top three FAR1 gene structures

**Figure 2.**
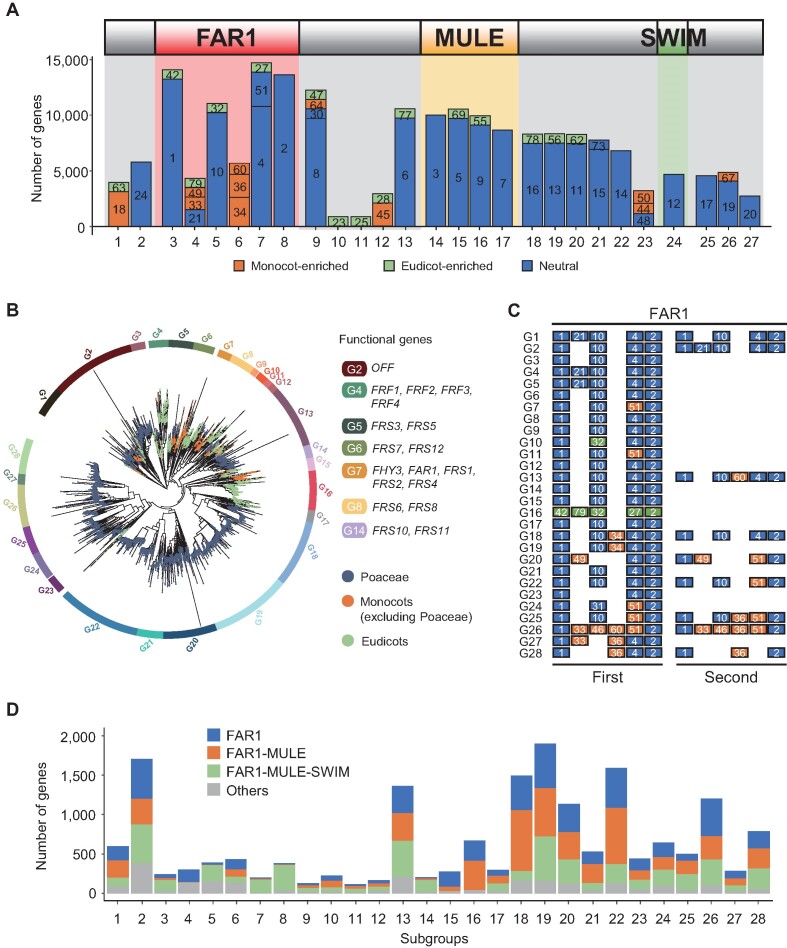
Motif composition of the top three domain architectures, phylogenetic relationships, and subgroup characteristics of FAR1 genes. (**A**) The motif architectures of the top three gene architectures, consisting of FAR1, MULE and/or SWIM domains, are portrayed. The numbers in bars indicate motif labels described in Supplementary Table S5. (**B**) The inferred phylogenetic relationships of FAR1 genes among 80 plant species are depicted. Colored bars on the outer ring show distinct subgroup divisions and colored dots on the branch tips indicate taxonomic groups. Functional FAR1 genes in *Oryza sativa* ssp. *japonica* and *Arabidopsis* are highlighted in the legend. (**C**) First and second most abundant motif compositions of FAR1 domains are outlined for each subgroup. The top two structures are shown when the number of the second most common structure was above 50. (**D**) The numbers of top three gene structures and other structures are shown for each subgroup. For panels A, C and D, box colors indicate monocot-enriched, eudicot-enriched or neutral (*P* < 0.0001).

For the three major gene structures of FAR1 genes, we identified and determined positions of 51 conserved motif sequences, excluding 28 motifs located repetitively in the N- and C-terminals of 16,210 F, FM or FMS genes (86%) (Figure 2A and Supplementary Table S5). Motifs in the region encompassing the MULE and SWIM domains were highly conserved, consistent with previous studies reporting high sequence similarity in the central transposases and C-terminal zinc-binding domains (8,11) (Figure 2A). In contrast, FAR1 domains were variable and included monocot-specifically expanded motifs adjacent to position 5, especially in the *Poaceae* family (98%), and eudicot-specifically expanded motifs in the *Fabaceae* family (64%), generating structural diversity in overall major FAR1 gene structures (Figure 2A and Supplementary Table S5). We next compared motif architectures of F, FM and FMS genes separately, revealing that their motif compositions were not distinct (Supplementary Figure S1), which suggests that all three major gene structures of the FAR1 repertoire presumably underwent structural changes after emerging from a common ancestor.

### Subgroup characteristics and phylogenetic relationships of FAR1 genes

We constructed a phylogenetic tree using 12,590 FAR1 protein sequences (67%) containing intact FAR1 domains with both start and end motifs (at positions 3 and 8) and determined all 18,744 FAR1 genes into 1 of 28 subgroups (Figure 2B; Supplementary Figures S2 and S3). Using another tree built with the same data, we validated the conservation of each clade with 99% overlap (Supplementary Figure S2). We observed specific subgroups of genes associated with distinct lineages; for example, 71% of genes in the *Poaceae* family, including 85% of wheat-related genes, composed a single large clade (99.9% of G18 to G28), indicating *Poaceae*-specific expansion, especially in species of wheat (Figure 2B; Supplementary Figure S3 and Table S6). Further examination revealed that the most abundant FAR1 motif architectures of each subgroup varied between subgroups. FAR1 genes in the *Poaceae*-expanded subgroups (G18 to G28) comprised monocot-enriched motifs, whereas the *G. max*-expanded G16 subgroup exhibited a eudicot-enriched FAR1 motif structure (Figure 2C and Supplementary Table S6). Moreover, we observed that most subgroups contained all three main gene structures, providing evidence that these gene structures originated from common ancestors of each subgroup and simultaneously underwent structural changes in different lineages (Figure 2D).

### Structural divergence of FAR1 genes via recurrent sequence mutations

Because genes do not likely simultaneously gain identical gene structures across different subgroups, we postulated that genes containing FM or F only underwent losses of MULE and/or SWIM domains through further evolutionary processes after emergence from their parental genes comprising FMS. To verify this hypothesis, we examined the downstream sequences of F and FM genes for evidence of residual MULE and SWIM domain sequences (Supplementary Figure S4). Of 5,559 F genes, 70% contained residual MULE and/or SWIM domains, whereas 37% of 5,634 FM genes contained remnants of SWIM domains, suggesting that those genes were altered to F or FM structures by relatively recent omission of MULE and/or SWIM domains (Supplementary Figure S4 and Table S7). When we analyzed the end motif positions of F and FM genes, most F (90%) and FM (74%) genes were predicted to be shortened due to premature translation termination (Supplementary Figure S5 and Table S8). These findings indicate that FAR1 gene structural changes mainly occurred via premature translation termination in F and FM genes, leaving traces of residual MULE and/or SWIM domains in downstream sequences.

**Figure 3.**
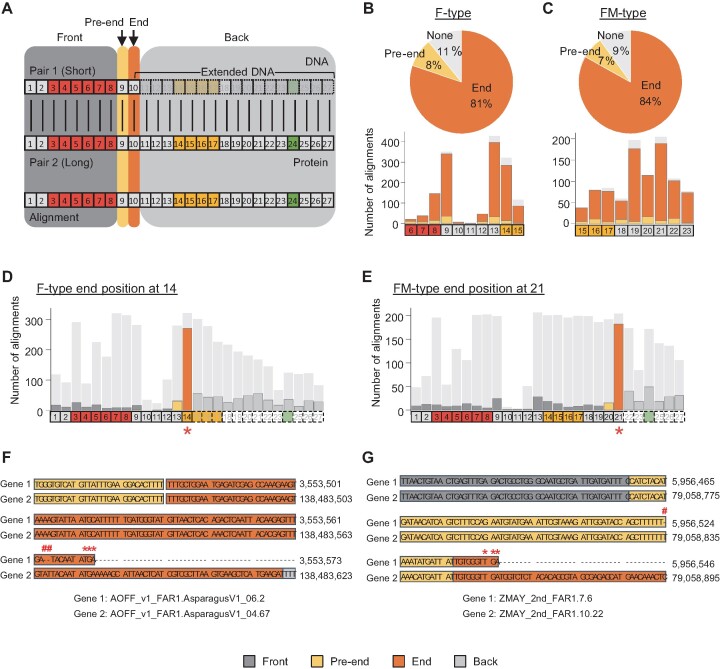
Distribution of accumulated frameshift or nonsense mutations in FAR1 (F-type) and FAR1-MULE (FM-type) pairs. (**A**) Motif positions of pairs and alignment result of the pair divided into four sections. Shorter and longer gene structures were designated Pair 1 and Pair 2, respectively. Motif containing the stop codon of Pair 1 was termed the ‘end’ motif site, and the motif directly upstream of ‘end’ was termed ‘pre-end’. Other motifs upstream of the ‘pre-end’ motif site were called ‘front’ motif sites, while motifs downstream of the ‘end’ motif site were called ‘back’ motif sites. Red, yellow and green boxes represent motifs of FAR1, MULE and SWIM domains, respectively. (**B**and **C**) The number of mutations in pre-end (yellow) or end (orange) motif sites for each set of alignments (light gray) with end motif sites at 6–15 for F-type and 15–23 for FM-type is illustrated as bar plots. The proportion of genes with mutation occurrences in the pre-end or end positions are shown as a pie chart. ‘None’ refers to alignments with no mutations in pre-end or end motif sites. (**D**and **E**) Specific examples show the mutation distribution in F- and FM-type alignments with end positions at 14 and 21, respectively. Light gray bars with no bar lines show the total number of motifs in each position of alignments while the colored bars indicate the number of alignments with mutations for each position. (B–E) The *x*-axis is the positional information of motifs. Red, yellow and green boxes represent motif positions of FAR1, MULE and SWIM domains, respectively. Solid boxes denote motif positions contained in the shorter pair, while dotted boxes are not part of the shorter pair but included in the alignments. Colors of bars delineate the motif position in relation to the end motif (asterisk) of the shorter pair. (**F**and **G**) Duplication pairs with a frameshift mutation within the end motif site or pre-end motif site causing premature termination are shown. Top genes are the shorter of the paired genes. Hashtag (#) represents the site where frameshift mutation is predicted and asterisks show the stop codon where premature termination occurred. Colors indicate the motif position in relation to the end motif site (orange) of the shorter pair.

To investigate the cause of premature translation termination of F and FM genes, we identified recent duplication pairs and classified them into two groups based on whether their domain structures were the same (60%) or different (40%) (Supplementary Figure S6). The latter duplication pairs were aligned to verify what caused the premature translation termination of shorter genes (denoted the ‘Pair 1’ sequence of each pair) containing F or FM compared to longer genes (‘Pair 2’ sequence of each pair) with FM or FMS (Figure 3A). Of the F and FM genes shortened by premature translation termination, 44% F (F-type) and 41% FM (FM-type) genes had a duplication pair with a different gene structure, of which 72% and 68% aligned pairs, respectively, remained after noise filtration and were used to examine sequence differences between Pair 1 and Pair 2 (Supplementary Figures S5B, S7 and Table S8). We next determined the number of mutations in alignments with end motif sites at 6 to 15 and 15 to 23 for F- and FM-type genes, respectively (Figure 3A–C). Our analyses revealed that frameshift and nonsense mutations causing premature translation termination were mostly accumulated at the end motif sites of both F- (81%) and FM-type (84%) genes, whereas 9% F- and 7% FM-type genes had mutations in their pre-end motif sites (Figure 3B and C). Specifically, F- and FM-type genes with end motif sites at 14 and 21, respectively, exhibited the same pattern, with 95% of F- and 97% of FM-type pairs containing frameshift or nonsense mutations at pre-end (9%) or end (87%) motif sites (Figure 3D and E). These findings demonstrate that mutations specifically accumulated at the end motif sites, together with those at pre-end motif sites, caused premature termination of translation in Pair 1 genes, generating a different gene structure from that of Pair 2 (Figure 3F and G).

Examination of the end motif site also revealed that 577 (10%) and 1,482 (26%) of total F and FM genes, respectively, had stop codons located downstream of MULE and/or SWIM domain regions but no MULE and/or SWIM domains were annotated, suggesting that a small proportion of F and FM genes underwent structural transformation by MULE and/or SWIM domain truncation (Supplementary Figure S8A and Table S8). Of these, 45% had duplication pairs with different gene structures, of which 192 (73%) F- and 235 (35%) FM-type pairs after noise filtration were analyzed (Supplementary Figures S7, S8B and Table S8). Although 60% of MULE and SWIM regions contained frameshift or nonsense mutations for F- and FM-type genes, respectively, this mutation pattern was not as prominent as those seen in instances of premature translation termination (Supplementary Figure S8C and D). Further examination of these pairs revealed that mutations within or surrounding the truncated domains caused variations in exon-intron structures, thereby generating changes in overall gene structure (Supplementary Figure S8E and F). For example, we found that a nonsense mutation within the MULE domain of one pair caused partial truncation of the second exon in Pair 1, altering some of its MULE motifs to yield an intron region (Supplementary Figure S8E). Thus, the truncated MULE domain was not annotated in Pair 1. Analysis of another pair without a mutation in a missing MULE or SWIM domain revealed that a region flanked by nonsense mutations in the fourth exon was converted to an intron, together with the SWIM domain (Supplementary Figure S8F). As a result, the SWIM domain was removed from the annotation in Pair 1. Taken together, we conclude that these frameshift and nonsense mutations impair normal translation of downstream sequences and/or truncated domains and generate gene structure variation in the FAR1 gene repertoire of plants.

### Functional investigations of a newly annotated gene in rice

**Figure 4.**
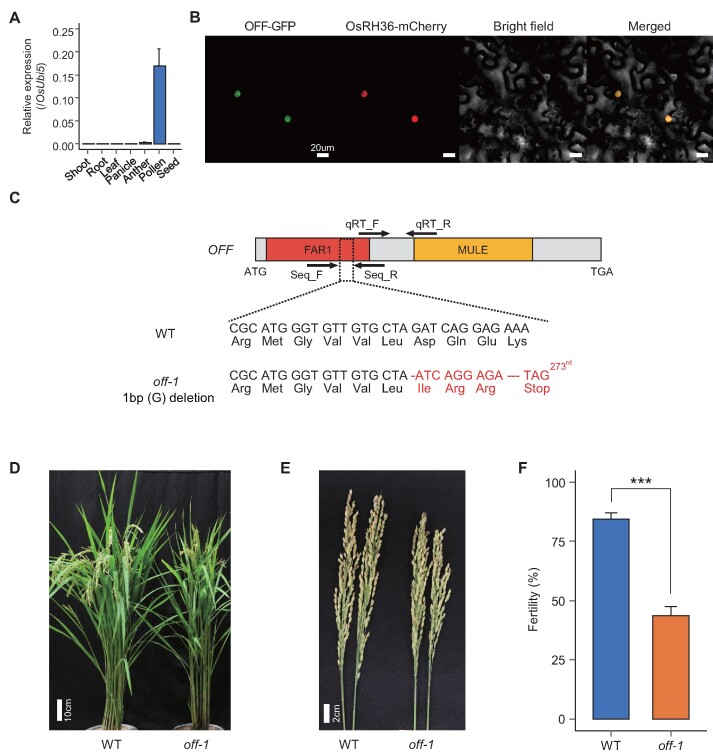
Functional investigation of the newly annotated FAR1 gene, *Oryza sativa FAR1 related to Fertility (OFF)*. (**A**) qRT-PCR analysis of *OFF* using seven rice tissues is illustrated. Relative expressions were calculated using the rice ubiquitin 5 gene (*OsUbi5*, *LOC_Os01g22490*). Three biological replicates were used for the analysis, and error bars indicate standard deviation. (**B**) Subcellular localization of the *OFF*-encoded protein in tobacco epidermal cells is shown. The OFF-GFP protein localized to the nucleus, merging exactly with the nuclear marker protein, OsRH36-mCherry (scale bar = 20 μm). (**C**) Gene structure of *OFF* and the sequence of its knockout mutant, *off-1*. The CRISPR/Cas9-targeted region is shown by dotted lines. Black arrows indicate primer regions for qRT-PCR and genotyping of the *off-1* mutant. WT represents wild-type plant (*O. sativa* ssp. *japonica* cv. Dongjin). (**D**and **E**) Phenotypes of the WT and *off-1* mutant plants during the ripening stage. (**F**) Fertility of the WT and *off-1* mutant plants. Five panicles from each of three replicates were analyzed. Error bars indicate standard deviation. *** means *P* < 0.001.

Because functional studies of FAR1 genes in monocots have been limited despite monocots containing the largest number of FAR1 genes, we verified the functionality of newly annotated FAR1 genes in *O. sativa* ssp. *japonica* (Supplementary Figures S9 and S10). Since the G2 subgroup contains the largest rice FAR1 genes, we investigated the expression patterns of target genes using the phylogenetic heatmap approach employed at the CAFRI-Rice website (http://cafri-rice.khu.ac.kr/) (27). *Oryza sativa FAR1 related to Fertility* (*OSAT.v7_FAR1.Chr10.2* and GenBank accession number MW602302; hereafter, referred as *OFF*) gene, a locus with an FM and aligned in the G2 subgroup, showed unique pollen-specific expression, suggesting that its function may be predominantly associated with rice pollen (Supplementary Figure S9A). We further confirmed its specific expression in pollen through quantitative RT-PCR analyses of the *OFF* in seven tissues (Figure 4A). Moreover, we performed a tobacco infiltration assay to determine its subcellular localization in the nucleus as a functional transcription factor (TF) (Figure 4B). These results demonstrate that the *OFF* is a preferentially pollen-expressed gene whose product localizes to the nucleus, showing exact overlap with the nucleus marker protein OsRH36-mCherry (46) (Figure 4A and B).

Using CRISPR/Cas9 genome editing, we generated a 1-bp deletion homozygous knockout mutant with a premature stop codon in its FAR1 domain (referred to as *off-1*) (Figure 4C). We observed that *off-1* plants in the T1 generation experienced reduced fertility to approximately 40% of that in wild-type plants but showed normal anther and pollen development during pollen maturation, indicating that *OFF*, which shares distinct domain architecture with known functional FAR1 genes, could encode a TF expressed in rice pollen that specifically affects fertility (Figure 4D–F and Supplementary Figure S9B). Furthermore, our results demonstrate that *OFF* could be a novel genetic resource for regulating fertility, unlike previously reported male sterility genes, which are associated with abnormal tapetum degradation and severely retarded anther and pollen development (47).

**Figure 5.**
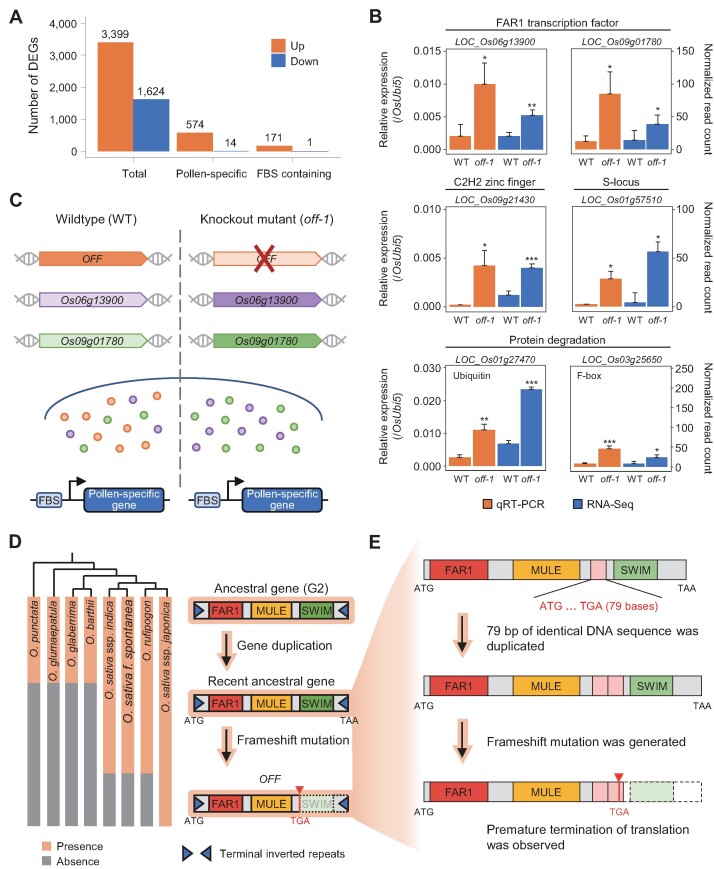
Transcriptional regulation of rice pollen by the mutation of *Oryza sativa FAR1 related to Fertility (OFF)* gene and its evolutionary history**. (A**) The number of differentially expressed genes (DEGs) is shown as a bar graph. FBS indicates FAR1 binding site (CACGCGC). Colors of bars show upregulated (orange) and down-regulated (blue) genes. (**B**) qRT-PCR validation of up-regulated FAR1 transcription factors and four regulatory genes. Relative expression values (orange) and normalized RNA-seq read counts (blue) are shown. The rice ubiquitin 5 gene (*OsUbi5, LOC_Os01g22490*) was used to calculate the relative expression value as an internal control. The error bars indicate standard deviation. * means *P* < 0.05; ** *P* < 0.01; *** *P* < 0.001. (**C**) Hypothetical model of *OFF* function in rice pollen. Colored dots indicate translated *OFF* (orange), *Os06g13900* (purple) and *Os09g01780* (green). Opaque colors represent high expression while lighter colors indicate low expression of genes. (**D**) The global evolutionary mechanism is illustrated. The recent parent gene derived from an ancestral FAR1 gene, common to all *Oryza* species in Group 2, was duplicated in *O. sativa*. Orange stripes on the left highlight *Oryza* subspecies that share evidence of the target gene shown on the right, while gray stripes indicate absence of such gene evidence. (**E**) The specific evolutionary process of *OFF* is depicted. After duplication of the recent parent gene, a 79-bp DNA sequence was duplicated, generating a frameshift mutation. Premature termination of protein translation (red arrowhead) was observed, and a function was newly acquired.

To explore the transcriptional regulation associated with *OFF*, we performed RNA-seq analysis using anthers at stages 13–14 (48) from the wild-type and *off-1* mutant plants. Subsequently, we identified 5,023 differentially expressed genes (DEGs) from the transcriptome analysis and elucidated 588 pollen-specific DEGs with more than a 2-fold change in expression between the wild type and *off-1* mutant (*P* < 0.05) (Figure 5A). Of these, 574 genes (97.6%) were upregulated in the *off-1* mutant, and most DEGs were enriched for functions associated with transcription regulation and protein degradation pathways (Figure 5A and Supplementary Figure S11). Notably, there were six E3 ligases in the protein degradation category (Supplementary Table S11). Although there have been no reports of the detailed protein degradation mechanism related to late pollen development in rice, there were reports that GORI and OsMTD2, key regulators in rice late pollen development, interact with the E3 ligase involved in protein degradation (42,49). Furthermore, we performed the yeast two-hybrid screening with FAR1 domain of the OFF protein and identified six interacting partners (Supplementary Figure S12). Among them, elongation factor 1 alpha showed the strongest interaction with the FAR1 domain among the interactors and there was a report that gene encoding this protein has a functional role in pollen development via participating in pollen sequestrome in tobacco pollen development (50). Thus, these downstream genes can be used as an important resource for further studies.

Because FAR1 proteins are known to specifically bind the FAR1-binding site (FBS, CACGCGC) of various target genes in *Arabidopsis* (8,51), we inspected 2-kb sequences upstream of each of the 574 upregulated DEGs and found that 29.8% of these genes contain FBS (Figure 5A). Of these, upregulation of two FAR1 TFs and four genes related to transcription regulation and protein degradation was identified in the *off-1* mutant (Figure 5B), suggesting a possibility of genetic compensation among homologous genes to alleviate severe phenotypes in a loss of function mutant for a gene as previously reported (52). Functional conservation among rice homologous genes expressed in reproductive tissue has also been reported (53,54). As another possibility, the *OFF* might act as a suppressor for FBS containing genes so that FBS genes are more expressed in the *off-1* mutant. Collectively, loss of function of *OFF* in the *off-1* mutant is suggested to increase the expression of genes with FBS sequences in the promoter region (Figure 5B and C). This is the first report of the *OFF* as a functional FAR1-associated TF that participates in transcriptional regulation within rice pollen, together with other FAR1 genes. We expect that our annotations and analyses of the *OFF* and those of FAR1 genes from the 80 queried plant species will be useful resources for characterizing late pollen development and other related adaptations in plants.

### Evolutionary adaptation of *OFF gene*

To illustrate how this functional gene emerged and ultimately adapted in *O. sativa* ssp. *japonica*, we first predicted the original structure of the most recent ancestral gene of the *OFF* via protein mapping using FMS genes of *Oryza* spp. in the G2 subgroup (Figure 5D and Supplementary Figure S13A). We detected recent ancestral gene structures containing FMS and two exons in only four *Oryza* spp.; however, all carried sequence mutations or deletions in different regions that generated translation errors (Figure 5D and Supplementary Figure S13A). This finding suggests that the recent ancestral gene emerged from the four *Oryza* spp. after their divergence from other *Oryza* spp. and subsequently lost the original gene structure by subspecies-specific sequence diversification (Supplementary Figure S13A). A detailed sequence alignment of these ancestral gene regions revealed that the *O. sativa* ssp. *japonica* genome contains two copies of a 79-bp sequence, whereas other *O. sativa* spp. have only one copy each, indicating that a tandem duplication of the 79-bp sequence occurred only in the recent ancestral gene of *O. sativa* ssp. *japonica* (Supplementary Figure S13B). This tandem duplication introduced a frameshift mutation in downstream sequences of the MULE domain of the recent ancestral gene of *O. sativa* ssp. *japonica*, leading to premature translation termination; this change illustrates how the *OFF* was shortened from its recent ancestral gene length and ultimately obtained the FM structure (Figure 5E). Thus, our results unveiled a novel adaptation mechanism involving structure truncation acquired through divergent evolution via frameshift mutation and the acquisition of a new functional gene that promotes fertility in *O. sativa* ssp. *japonica* (Figure 5E).

## CONCLUSION

It is unclear how TE-derived genes that generally exist as high-copy numbers in plant genomes have diversified and adapted after their initial emergence. In this study, we aimed to understand the underlying adaptation process promoting structural and functional divergence of FAR1 genes based on comprehensive comparative studies using improved annotations of the MULE-derived TF family. We discovered a large pool of newly identified genes, especially in the *Poaceae* family of monocots, most of which formed *Poaceae*-enriched clades with monocot-specific FAR1 domains, suggesting that these genes may have evolved to meet specific demands of monocots in a different growth environment. Moreover, despite many known functional genes containing FMS structures in *Arabidopsis*, our data revealed that FM structures were the most prevalent and may serve as a new source of TFs that control agriculturally important traits in plants. Previous studies often focused on the emergence and adaptation mechanism of individual functional genes with little or no insight into a global picture on a genomic scale (55). By comparing the top three gene structures of FAR1 in 80 plant species, we revealed that an accumulation of frameshift and nonsense mutations causing premature translation termination in ancestral FMS genes was a key evolutionary mechanism in continuously generating widespread structural divergence of FAR1 genes. Although many of these genes may have become inactive under negative selection, several genes driven by positive selection could have obtained species-specific functions. Through CRISPR/Cas9-based genome editing approach, we unveiled a novel functional gene with an FM structure in rice that regulates fertility-associated transcription in pollen, together with other FAR1 TFs which validates our gene models as practical resources for comprehensive functional investigations of important agricultural crops. Other FAR1 TFs seemed to compensate for the loss of function for *OFF*, suggesting sub-functionalization of duplicated FAR1 genes in this multigene family (56). Closer inspection of the novel functional gene illustrated that this gene obtained its shortened form through a species-specific frameshift mutation and finally acquired an agriculturally important trait. Through a combined approach integrating computational analysis with molecular work, we explored a fundamental evolutionary mechanism underlying adaptation by natural selection. Taken together, our findings unveil a key evolutionary mechanism that contributes to widespread structural divergence and subsequent functional adaptation of MULE-derived TF genes in plants.

## DATA AVAILABILITY

The gene sequence of *OFF* was uploaded to GenBank under the accession number MW602302.

## Supplementary Material

gkab932_Supplemental_Files
