# Supplementary material for: Recurrent mutations promote widespread structural and functional divergence of MULE-derived genes in plants
Source: Nucleic Acids Res. 2021 Nov 2;49(20):11765–77. doi: 10.1093/nar/gkab932 (PMC8599713; doi:10.1093/nar/gkab932)
Supplement: gkab932_Supplemental_Files [file gkab932_supplemental_files.zip › 20210908_NAR_FAR1_supplementary_figures_revised_update.pdf]

Supplementary Figure 1

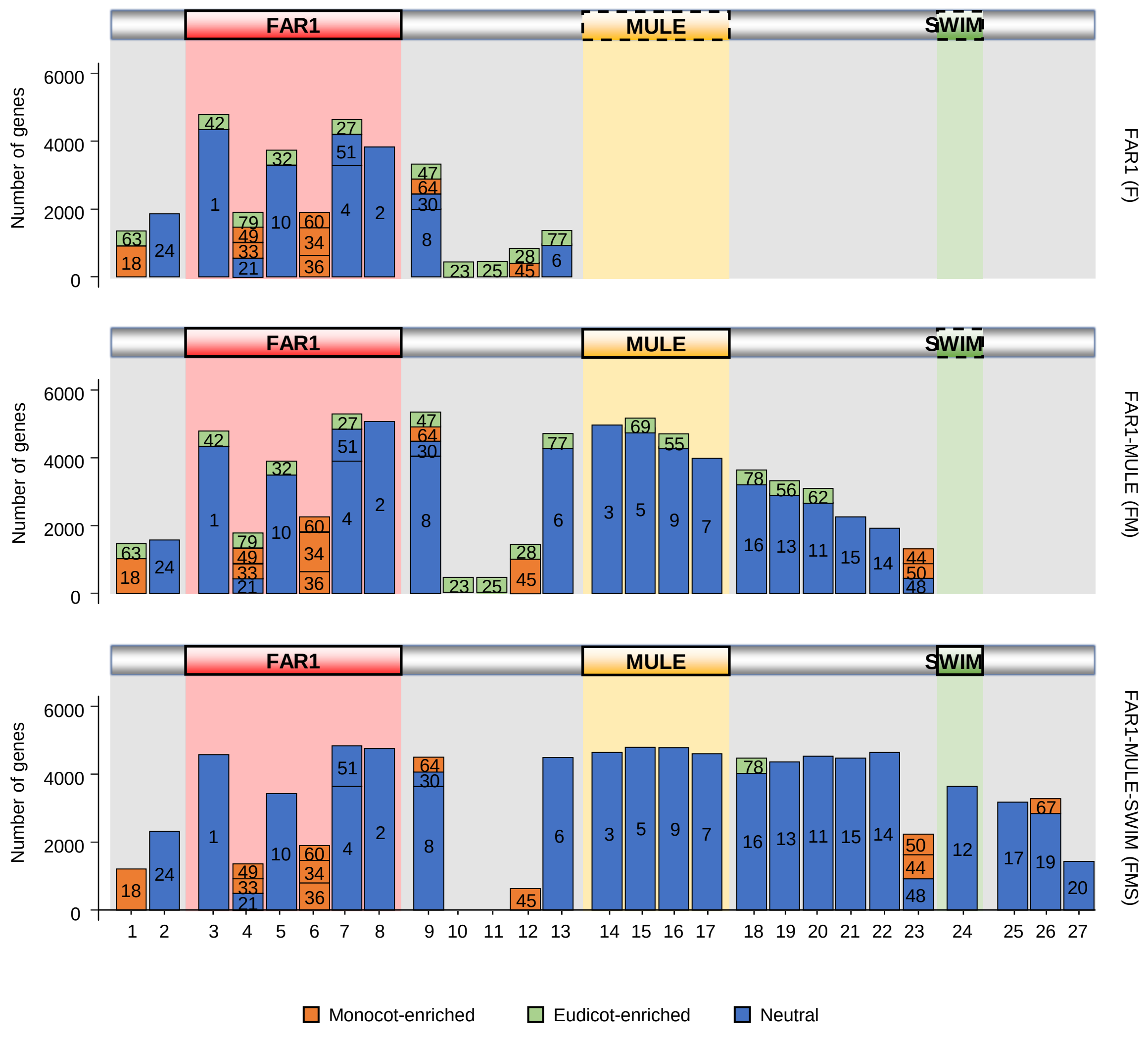

Supplementary Figure 1. Motif composition of top three gene structures in FAR1 genes. The x-axis is the motif position and y-axis is the number of genes containing each motif shown in bars. Bars were colored according to chi-square test showing whether each motif is monocot-enriched (orange), eudicot-enriched (green), or not enriched in a particular lineage (blue). Red, yellow, and green regions represent FAR1, MULE, and SWIM domains, respectively, while gray regions are sequences in between major domains. Domains shown in dotted boxes are absent from the gene structure.

Supplementary Figure 2

A

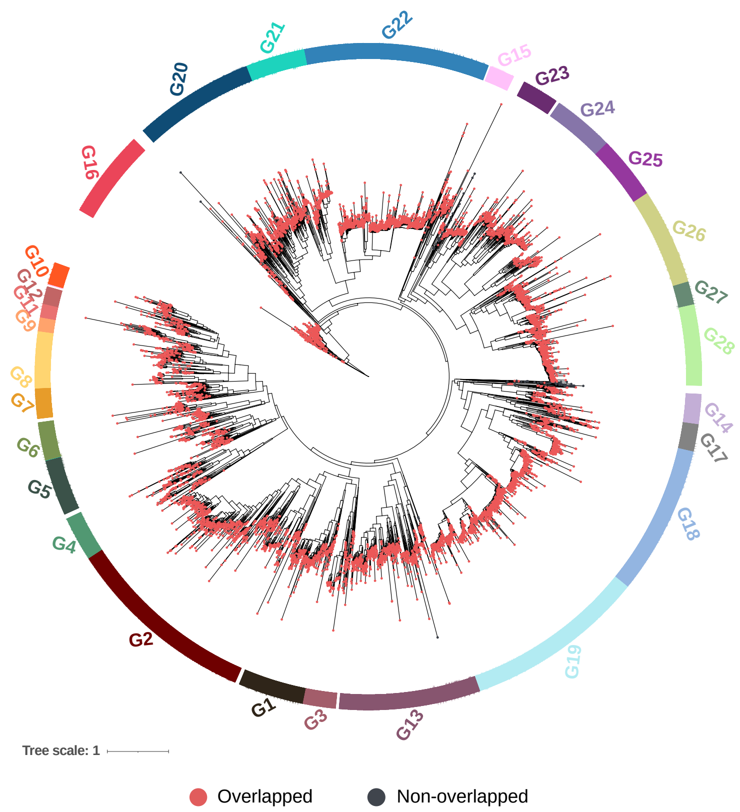

B

Group	Overlap (%)
G1	99.76
G2	99.92
G3	100
G4	100
G5	100
G6	80.13
G7	100
G8	100
G9	100
G10	100
G11	100
G12	100
G13	99.21
G14	98.4
G15	87.15
G16	99.64
G17	100
G18	100
G19	100
G20	99.87
G21	100
G22	100
G23	100
G24	100
G25	100
G26	100
G27	100
G28	100
**Total**	**99.22**

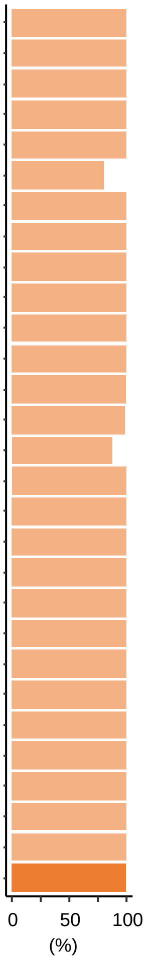

Supplementary Figure 2. Phylogenetic tree constructed using IQ-TREE and the corresponding overlapping ratios with the tree described in Figure 3. (A) Phylogenetic relationships of FAR1 genes with intact FAR1 domains are shown. The outer bar represents subgroups, while colored dots at branch tips show if the branch is in the same subgroup as previously assigned (red) or not (black). **(B)** The table shows what percentage of each subgroup is overlapping with the subgroups defined in the tree in Figure 3. The orange bars on the right visualizes the percentages.

Supplementary Figure 3

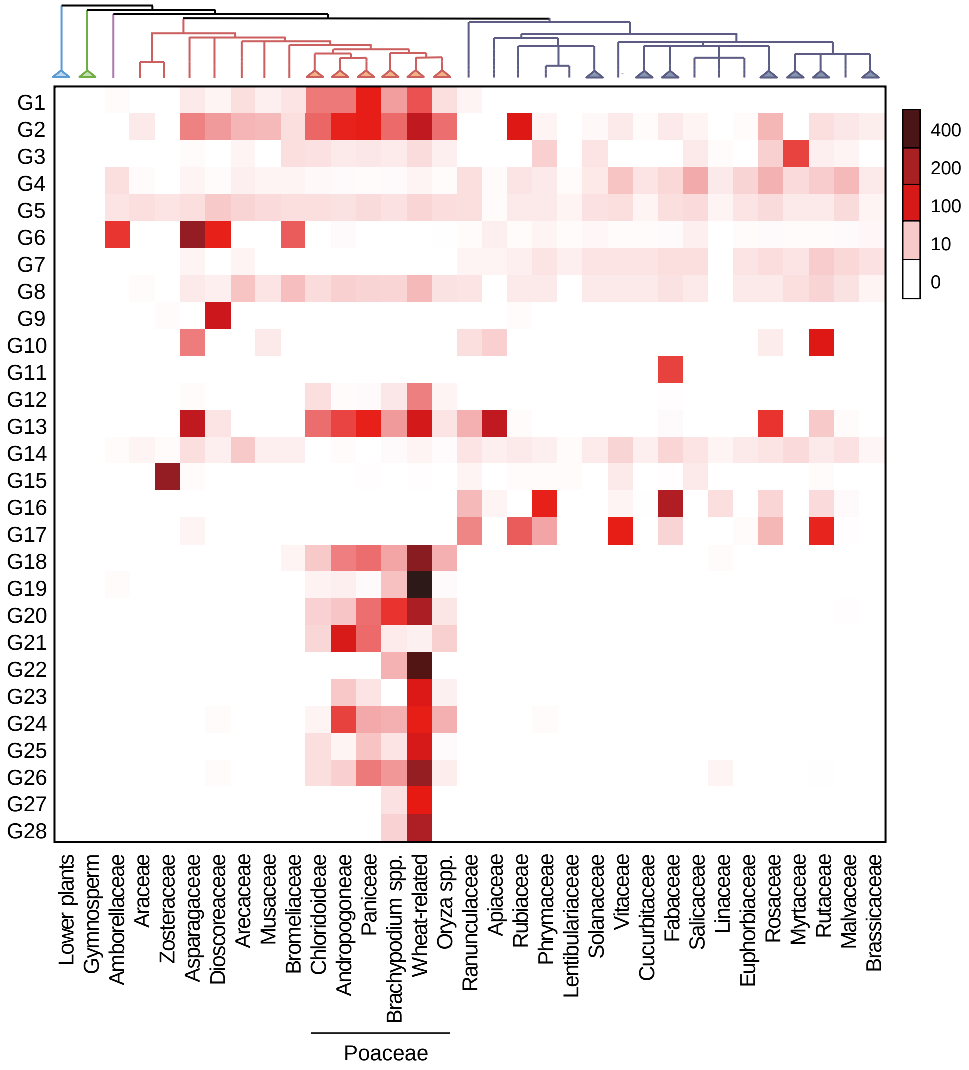

Supplementary Figure 3. The number of FAR1 genes in each family contained in each subgroup. The colors represent the number of genes as shown in the legend. The phylogenetic relationships between families are shown on top, and the colors of branches represent lower plants (blue), gymnosperms (green), monocots (red), and eudicots (purple).

Supplementary Figure 4

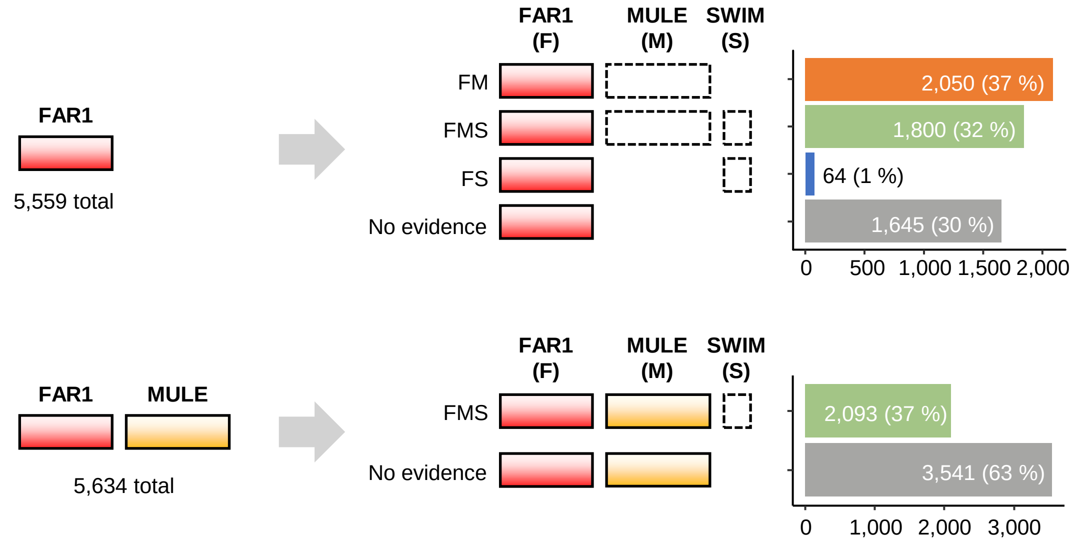

Supplementary Figure 4. Evidence of residual MULE and/or SWIM domains in downstream sequences of F and FM genes. Dotted boxes represent domains with evidence. Color of bars represent different gene structures with downstream evidence of MULE and/or SWIM domains, while gray bars show number of genes with no evidence.

Supplementary Figure 5

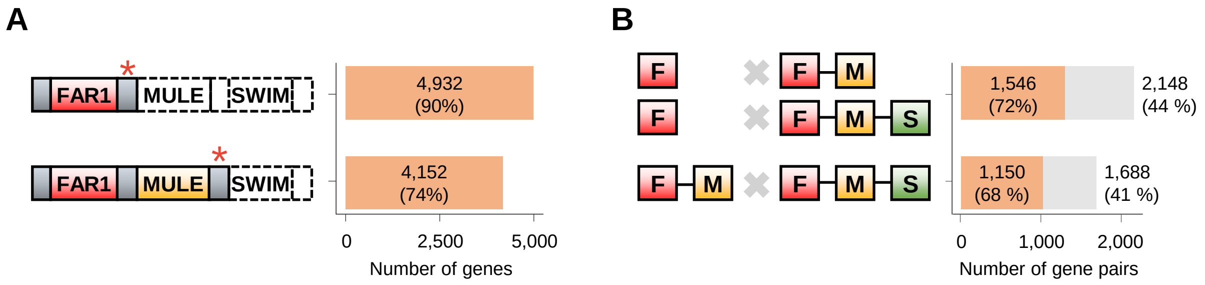

Supplementary Figure 5. Number of FAR1 (F-type) and FAR1-MULE (FM-type) pairs shortened by premature translation termination. (A) The numbers of putative F and FM genes altered by premature translation termination are shown. **(B)** Duplication pairs containing F and FM genes shortened by premature translation termination were counted (gray), of which the number of clearly aligned pairs are highlighted (orange).

Supplementary Figure 6

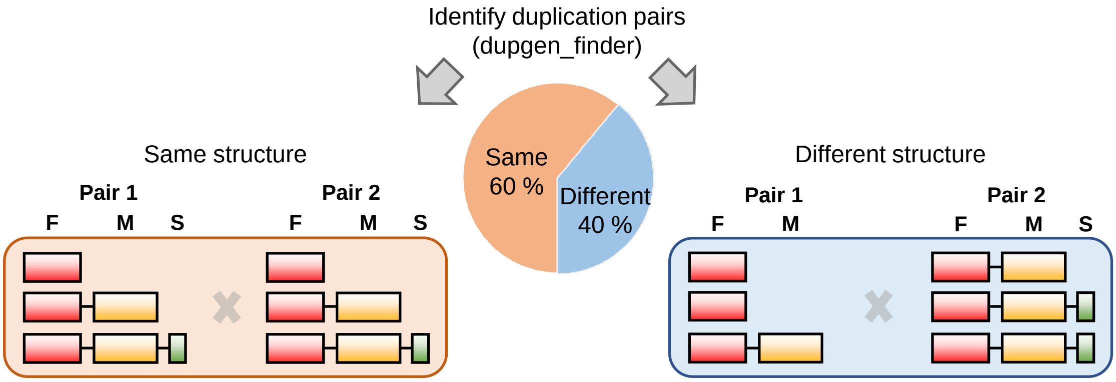

Supplementary Figure 6. Proportion of duplication pairs with same or different gene structures and motif positions of pairs and alignment result of the pair divided into four sections. Of pairs consisting of top three gene structures, pairs with same gene structures are shown on the left in an orange box, while pairs with different gene structures are shown on the right in a blue box.

Supplementary Figure 7

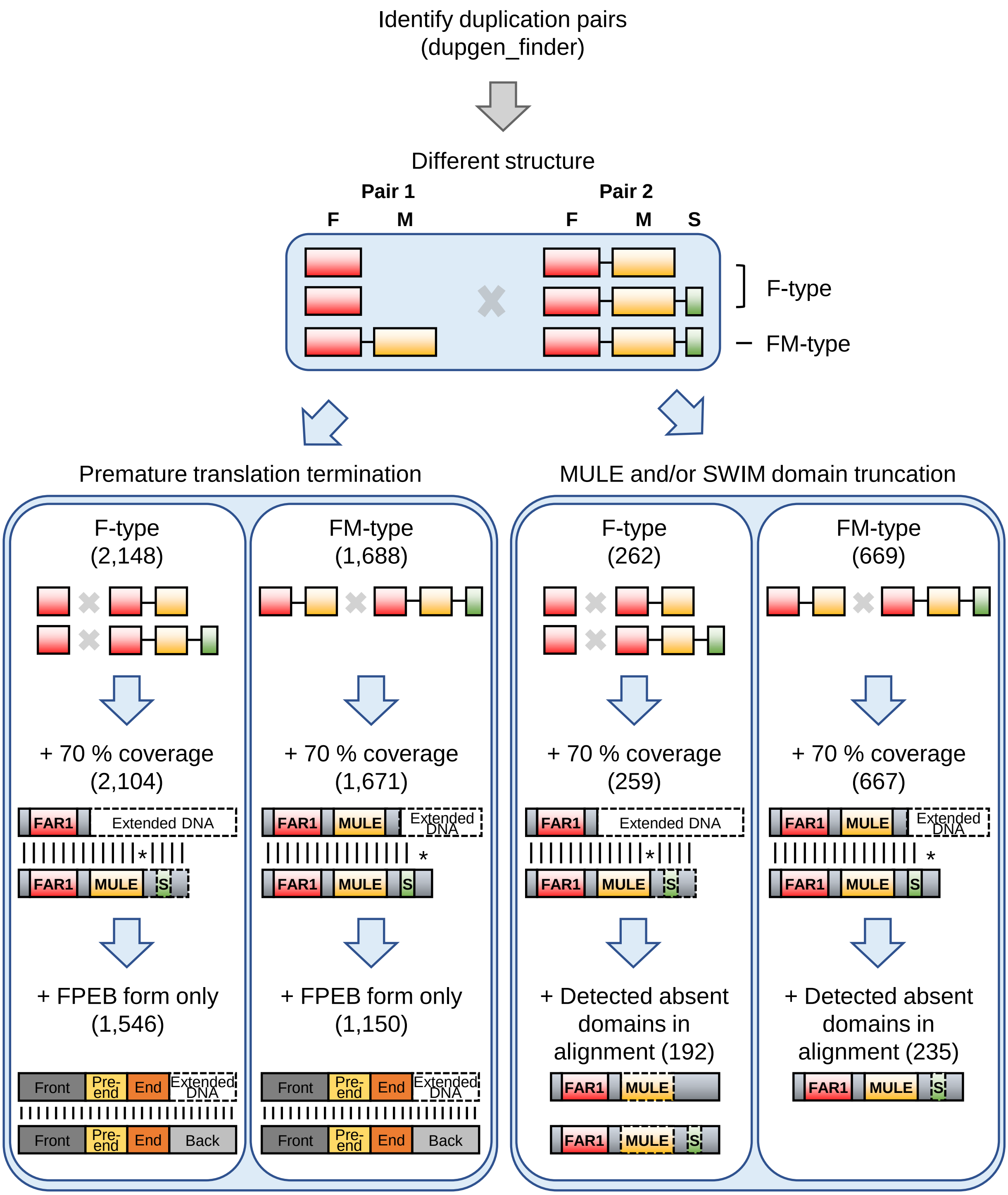

Supplementary Figure 7. Noise filtration processes of duplication pairs with different gene structures for further downstream analyses. Sequence variation for premature termination and MULE and/or SWIM domain truncation were viewed separately. Duplication pairs were separated into F-type and FM-type depending on the structure of Pair 1. Alignments mapping less than 70% of Pair 2 sequences were filtered. For premature termination, alignments containing all four sections (front, pre-end, end, and back) were considered for analysis, while for domain truncation, alignments with evidence of absent domains were considered.

Supplementary Figure 8

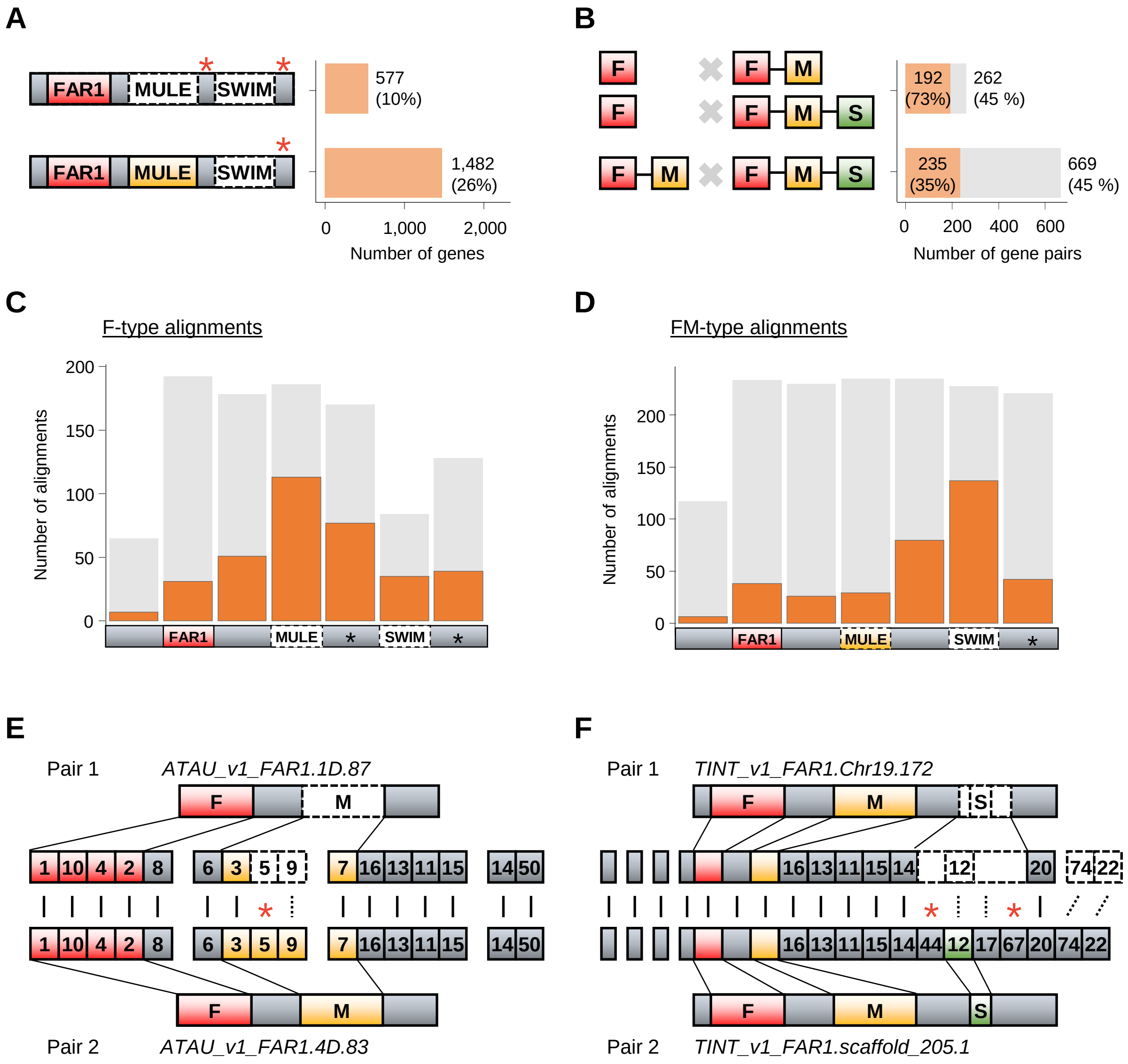

Supplementary Figure 8. Distribution of accumulated frameshift or nonsense mutations in FAR1 (F-type) and FAR1-MULE (FM-type) pairs with MULE and/or SWIM domain truncation. **(A)** The numbers of putative F and FM genes altered by MULE and/or SWIM domain truncation are shown. **(B)** Duplication pairs containing F and FM genes shortened by MULE and/or SWIM domain truncation were counted (gray), of which the number of clearly aligned pairs are highlighted (orange). **(C-D)** The number of mutations in each domain and inter-domain region are shown for F- and FM-types. Light gray bars with no bar lines show the total number of alignments containing each region. **(E-F)** Specific examples of wheat-related species, *A. tauschii* and *T. intermedium* respectively, show that mutation alters exon-intron structures, causing truncation of MULE and/or SWIM domains in Pair 1 sequences. Red, yellow, and green boxes represent motifs of FAR1, MULE, and SWIM domains, respectively. Solid boxes are motifs or domains contained in each pair, while dotted boxes are motifs or domains included in the alignment but not contained in Pair 1.

Supplementary Figure 9

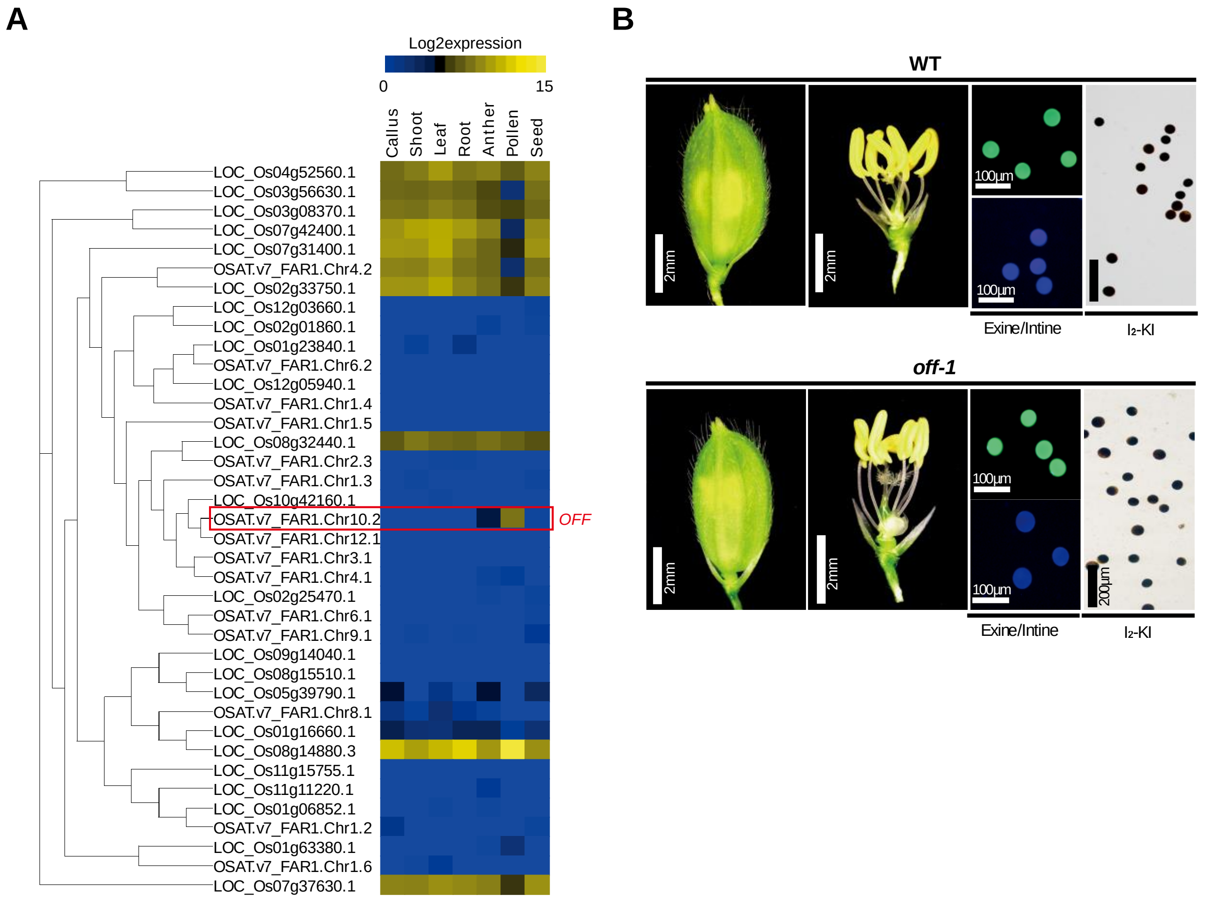

Supplementary Figure 9. Phylogenetic heatmap of FAR1 genes in the G2 subgroup and the phenotype of spikelet, anther, and pollen of the wild-type plant and *off-1* mutant. (A) The red box highlights the unique pollen-specific expression of *OFF*. The log2expression on the color bar means log2 transformed normalized read counts. **(B)** Besides spikelet and anther morphology, pollens stained using 1 % I_2-KI, 0.1 % calcofluor white, and 0.001 % auramine O solution showed no difference between the wildtype and the *off-1* mutant. Bar indicates 2mm.

Supplementary Figure 10

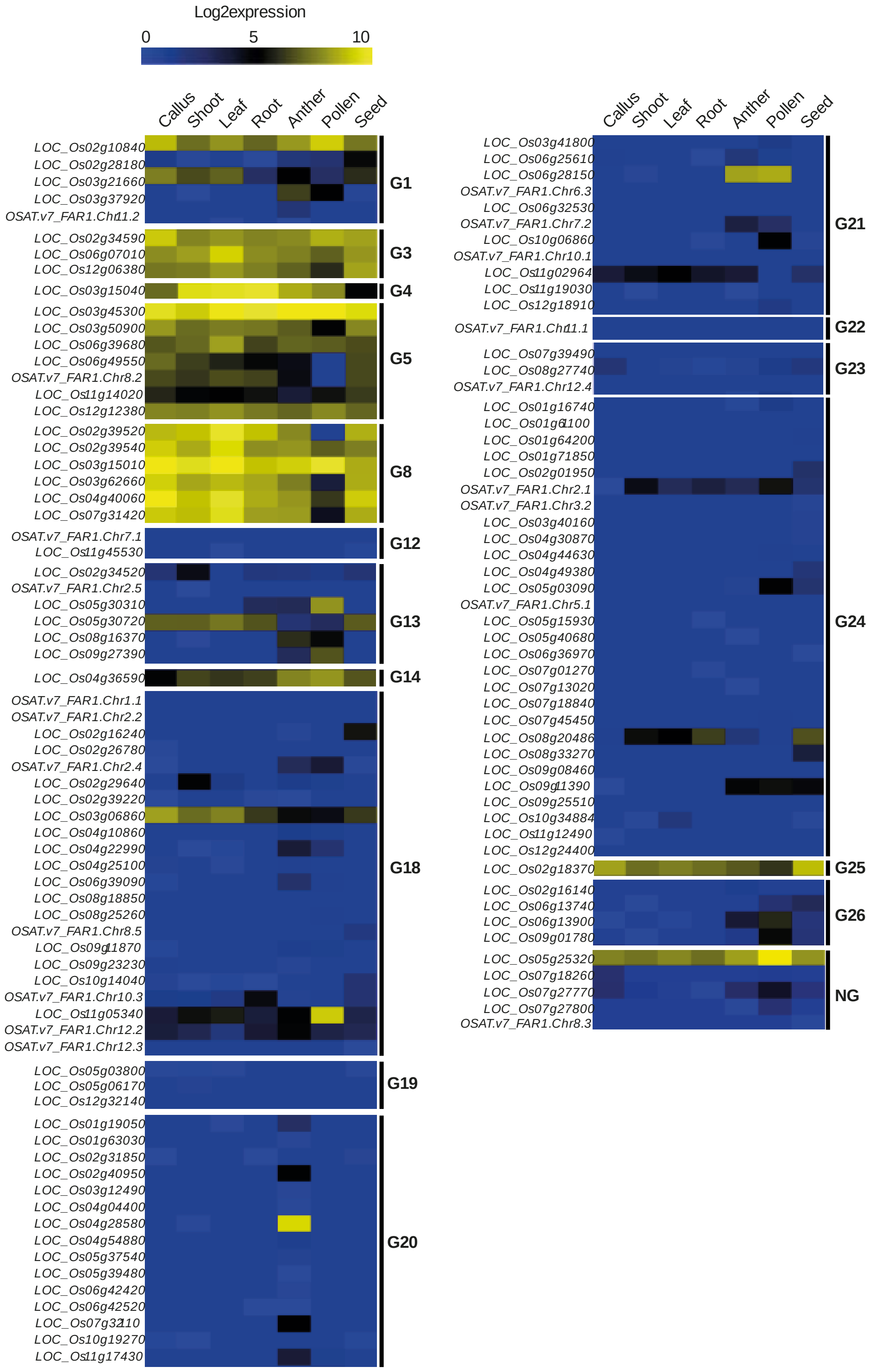

Supplementary Figure 10. Heatmap analysis of rice genes in other subgroups (except subgroup G2). The 124 rice FAR1 genes classified in subgroups excluding G2 subgroup were visualized as heatmap. The log2expression on the color bar means log2 transformed normalized read counts. There were more than five FAR1 genes showing pollen-preferred expression pattern like *OFF* gene.

Supplementary Figure 11

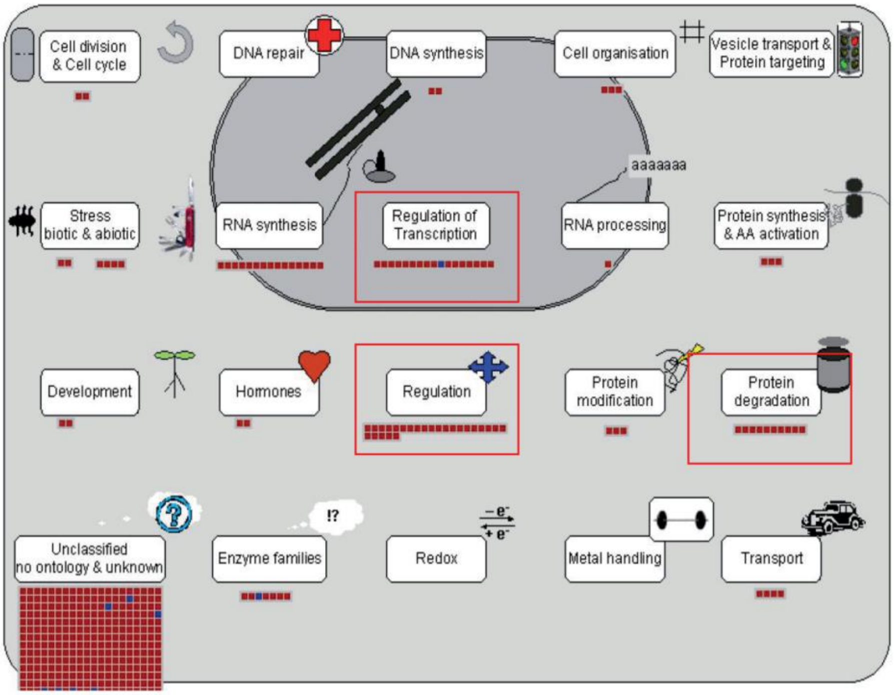

Supplementary Figure 11. Regulation overview of differentially expressed genes (DEGs) between the wild-type vs *off-1* mutant installed in MapMan software (41). Red and blue square indicate up or down-regulated DEGs respectively. Most DEGs are enriched in regulation, regulation of transcription, and protein degradation which imply loss of function of *OFF* affected transcriptional regulation of regulatory genes.

Supplementary Figure 12

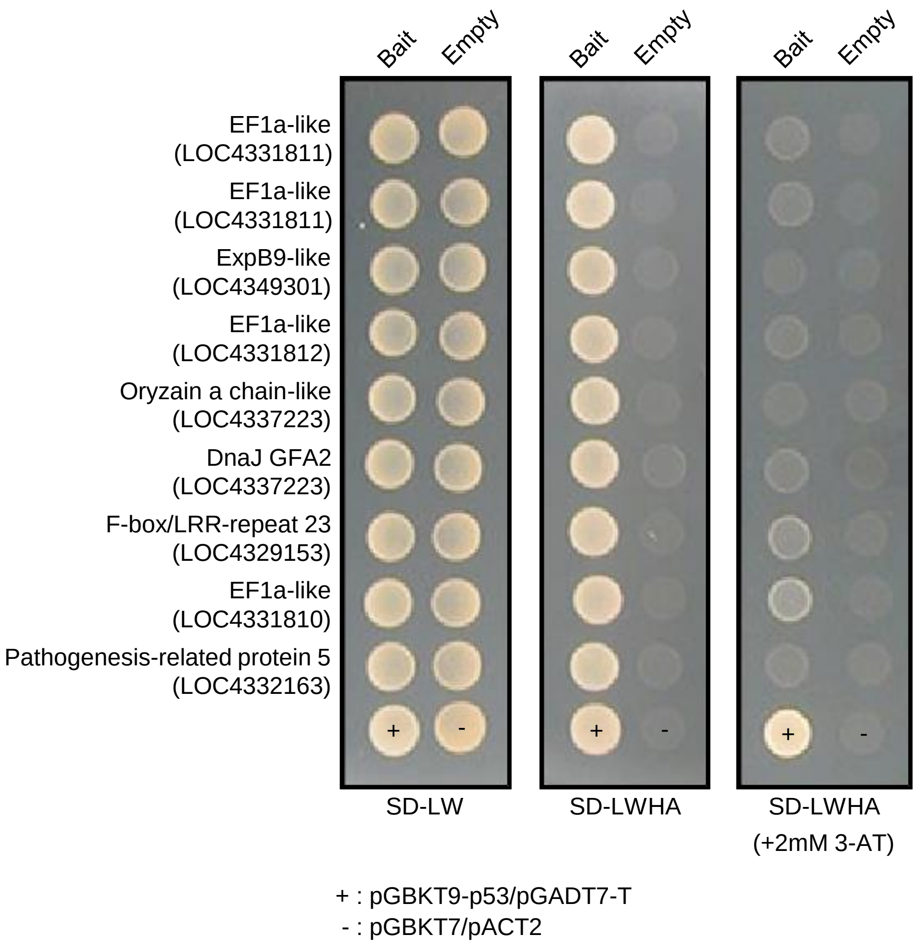

Supplementary Figure 12. Identification of interacting partners for FAR1 genes.
The FAR1 domain of *OFF* containing bait was used for yeast two-hybrid screening assay to find out interacting partner for FAR1 genes as previously described (42). Briefly, the yeast library was constructed from rice anther cDNA and plasmids of the bait and preys were transformed to the AH109 strain. SD minimal medium lacking leucine and tryptophan (SD-LW) and lacking leucine, tryptophan, histidine and adenine (SD-LWHA) were used as a master plate and selection medium. 2mM 3-Amino-1,2,4-triazole (3-AT) were treated to show strength of these protein interactions. The pGBKT9-p53/pGADT7-T and pGBKT7/pACT2 were used as positive and negative controls, respectively.

Supplementary Figure 13

A

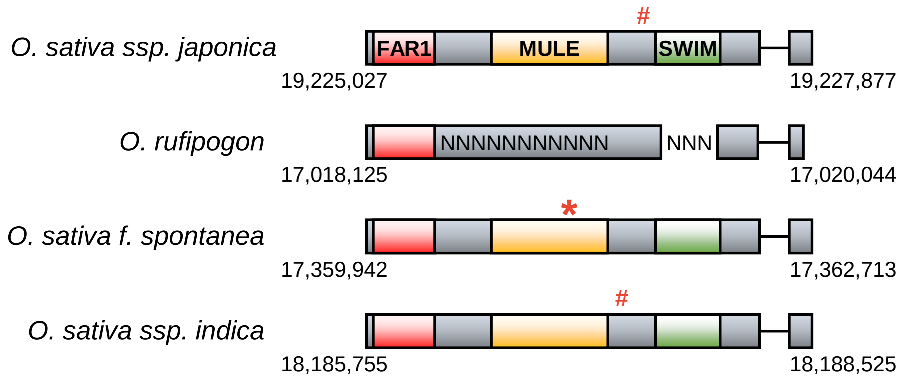

B

```
O. sativa ssp. japonica  AGAGAAAATG AGCTAAATGC TGAATTTGAA TCAAGGAAGA AAATACCCAG AATAAAAATG 19,226,344
O. rufipogon             ---------- ---------- ---------- ---------- ---------- ---------- 17,018,787
O. sativa f. spontanea   AGAGAAAATG AGCTAAATGC TGAATTTGAA TCAAGGAAGA AAATACCCAG AATAAAAATG 17,361,401
O. sativa ssp. indica    AGAGAAAATG AGCTAAATAC TGAATTTGAA TCAAGGAAGA AAATACCCAG AATAAAAATG 18,187,215

O. sativa ssp. japonica  AGGACACCTA TGCTAATCCA AGCTAGCAAG CTATACACAC CAATCATATT CGAAGCTTTT 19,226,404
O. rufipogon             ---------- ---------- ---------- ---------- ---------- ---------- 17,018,787
O. sativa f. spontanea   AGGACACCTA TGCTAATCCA AGCTAGCAAG CTATACACAC CAATCATATT CGAAGCTTTT 17,361,461
O. sativa ssp. indica    AGGACACCTA TGCTAATCCA AGCTAGCAAG CTATACACAC CAATCATATT CGAAGCTTTT 18,187,275

O. sativa ssp. japonica  CAAGCTGAAT ATGAAAGATC CATGGTAGCA TGCACCACGG CATTGGAAGG CAACAATTG  19,226,464
O. rufipogon             ---------- ---------- ---------- ---------- ---------- ---------- 17,018,787
O. sativa f. spontanea   CAAGCTGAAT ATGAAAGATC CATGGTAGCA TGCACCACGG CATTGGAAGG CAACAATTG  17,361,521
O. sativa ssp. indica    CAAGCTGAAT ATGAAAGATC CATGGTAGCA TGCACCACGG CATTGGAAGG CAACAATTG  18,187,335

O. sativa ssp. japonica  TATCTTGTGG CAATTGGCAG TCTAGATGAA AATTGTACCT ATGGTAGCAT GCACCACGG  19,226,524
O. rufipogon             ---------- ---------- ---------- ---------- ---------- ---------- 17,018,787
O. sativa f. spontanea   TATCTTGTGG CAATTGGCAG TCTAGATGAA AATTGTACCT ---------- ---------- 17,361,561
O. sativa ssp. indica    TATCTTGTGG CAATTGGCAG TCTAGATGAA AATTGTACCT ---------- ---------- 18,187,375

O. sativa ssp. japonica  ATTGGAAGGC AACAATTGCT ATCTTGTGGC AATTGGCAGT CTAGATGAAA ATTGTACCTT 19,226,584
O. rufipogon             ---------- ---------- ---------- ---------- ---------- ---------- 17,018,787
O. sativa f. spontanea   ---------- ---------- ---------- ---------- ---------- ---------T 17,361,562
O. sativa ssp. indica    ---------- ---------- ---------- ---------- ---------- ---------T 18,187,376

O. sativa ssp. japonica  TGAGAAGGAG TACAAAGTTG TTGGTGATCC TTTAGAGCAA ACTAGTACAT GCGGCTGTGG 19,226,644
O. rufipogon             ---------- ---------- ---------- ---------- ---------- ---------- 17,018,787
O. sativa f. spontanea   TGAGAAGGAG TACAAAGTTG TTGGTGATCC TTTAGAGCAA ACTAGTACAT GCGGCTGTGG 17,361,622
O. sativa ssp. indica    TGAGAAGGAG TACAAAGTTG TTGGTGATCC TTTAGAGCAA ACTAGTACAT GCGGCTGTGG 18,187,436

O. sativa ssp. japonica  GATGTTCAGT AGAACTGGAA TATTGTGTGC ACATGCTTTA AAAGTCCTTG ATTTGATGAA 19,226,704
O. rufipogon             ---------- ---------- ---------- --ATGCTTTA AAAGTCCTTG ATTTGATGAA 17,018,815
O. sativa f. spontanea   GATGTTCAGT AGAACTGGAA TATTGTGTGC ACATGCTTTA AAAGTCCTTG ATTTGATGAA 17,361,682
O. sativa ssp. indica    GATGTTCAGT AGAACTGGAA TATTGTGTGC ACATGCTTTA AAAGTCCTTG ATTTGATGAA 18,187,496
```

Supplementary Figure 13. Recent parental gene structures identified in *Oryza spp.* and 79-bp duplication specific to *O. sativa ssp. japonica*. (A) The gene structures of recent parental genes of *Oryza spp.* in G2 were predicted to contain two exons and FAR1, MULE, and SWIM domains (FMS). *O. rufipogon* consisted of three exons due to gaps and truncated sequences. The numbers are start and end positions of each gene in chromosome 10 of each species. Red, yellow, and green boxes represent FAR1, MULE, and SWIM domains, respectively. Approximate frameshift and nonsense mutation sites are shown by hashtags and asterisk, respectively. **(B)** *Oryza spp.* contained a 79-bp sequence which was tandem duplicated specifically in *O. sativa ssp. japonica.*
